# DLTKcat: deep learning-based prediction of temperature-dependent enzyme turnover rates

**DOI:** 10.1093/bib/bbad506

**Published:** 2024-01-06

**Authors:** Sizhe Qiu, Simiao Zhao, Aidong Yang

**Affiliations:** Department of Engineering Science, University of Oxford, OX1 3PJ, United Kingdom; Radcliffe Department of Medicine, University of Oxford, OX3 9DU, United Kingdom; Department of Engineering Science, University of Oxford, OX1 3PJ, United Kingdom

**Keywords:** deep learning, compound–protein interaction, enzyme turnover rate, temperature dependence, genome-scale metabolic modeling

## Abstract

The enzyme turnover rate, ${k}_{cat}$, quantifies enzyme kinetics by indicating the maximum efficiency of enzyme catalysis. Despite its importance, ${k}_{cat}$ values remain scarce in databases for most organisms, primarily because of the cost of experimental measurements. To predict ${k}_{cat}$ and account for its strong temperature dependence, DLTKcat was developed in this study and demonstrated superior performance (log10-scale root mean squared error = 0.88, *R*-squared = 0.66) than previously published models. Through two case studies, DLTKcat showed its ability to predict the effects of protein sequence mutations and temperature changes on ${k}_{cat}$ values. Although its quantitative accuracy is not high enough yet to model the responses of cellular metabolism to temperature changes, DLTKcat has the potential to eventually become a computational tool to describe the temperature dependence of biological systems.

## INTRODUCTION

In the age of synthetic biology, more and more chemical processes are being catalyzed by enzymes [1, 2], and therefore, the quantitative study of enzyme kinetics becomes an important topic. The enzyme turnover rate, ${k}_{cat}$, is one of the most important parameters in describing enzyme kinetics, which quantifies the maximum efficiency of an enzyme in catalyzing a specific reaction [3]. In spite of its importance, there currently exists a huge gap of measured ${k}_{cat}$ for most organisms in commonly used enzyme databases [4], i.e. BRENDA [5] and SABIO-RK [6]. Also, measuring ${k}_{cat}$ values via enzyme assays is expensive and labor intensive [4], which means that it is hard to obtain ${k}_{cat}$ values in a high-throughput manner. The limited availability of ${k}_{cat}$ in databases and the indispensable requirement for ${k}_{cat}$ in the study of enzyme kinetics and other fields, such as metabolic modeling [7], fuel the impetus behind the development of computational methods to predict ${k}_{cat}$ values.

There are two main methods to predict ${k}_{cat}$ values: (1) estimating ${k}_{cat}$ based on apparent catalytic rate (${k}_{app}$) with proteomic and fluxomic profiling and (2) predicting ${k}_{cat}$ using the compound–protein interaction (CPI) deep learning model. The first method obtains the ${k}_{cat}$ value by dividing the measured reaction flux by the quantified protein abundance [8, 9]. Although this method has been proved successful in resource allocation models of various microorganisms [10–13], fluxomics and proteomics are costly to measure, making this method difficult to implement.

CPI deep learning models have already been developed to predict biological parameters such as binding affinities (${K}_d$) [14], Michaelis–Menten constants (${K}_m$) [15] and enzyme turnover rates (${k}_{cat}$) [16]. The inputs are usually simplified molecular-input line-entry system (SMILES) strings of compounds and subsequences of proteins. Compound and protein features are extracted by graph neural network, recurrent neural network or convolutional neural network (CNN), and then concatenated for the regression of the target value, such as ${k}_{cat}$ or ${K}_m$ [17]. For better performance, attention layers are added to capture the interaction between compound and protein features [18, 19]. DLKcat [16], the first CPI deep learning model for ${k}_{cat}$ prediction, can predict $\mathrm{lo}{\mathrm{g}}_{10}\left({k}_{cat}\right)$ with the root mean squared error (RMSE) score below 1 and Pearson’s *r* = 0.71 for the test data set. However, one limitation of DLKcat and most other CPI models is that they do not account for experimental conditions like temperature, pH or ionic strength. As ${k}_{cat}$ has a strong dependence on temperature [20] and temperature is widely available in databases, developing a deep learning model that takes compound, protein and temperature features together as inputs are both necessary and approachable.

TurNuP [21], a CPI model for ${k}_{cat}$ with enhanced performance than DLKcat, included temperature as a feature in a case study to predict ${k}_{cat}$ for *Escherichia coli* (*E. coli*), but it was not a general predictive model for temperature-dependent ${k}_{cat}$. EF-UniKP and Revised UniKP [22] were developed to predict temperature-dependent ${k}_{cat}$ values. They considered ${k}_{cat}$ values at different temperatures and include the temperature value as a feature. However, the feature importance of temperature in those two models was not assessed, and no case studies were conducted to show the model’s ability to predict the effect of temperature on ${k}_{cat}$ values. Also, the *R*-squared (*R*^2^) scores of predictions by those two models were reported to be below 0.5.

With the aim to construct a deep learning model on ${k}_{cat}$ prediction that is more accurate than previously published models, this study developed DLTKcat. DLTKcat is a bidirectional attention CPI model with molecular graphs converted from SMILES strings, 3-mer subsequences of proteins and temperature features as inputs. It showed superior performance (log10-scale RMSE = 0.88, *R*^2^ = 0.66) than previously published models (e.g. EF-UniKP), and demonstrated the feature importance of temperature. Then, DLTKcat exhibited its potential application in enzyme sequence design by predicting the effect of amino acid substitutions on ${k}_{cat}$ at different temperatures. Finally, we incorporated temperature-dependent proteome constraints in bacterial metabolic modeling with predicted ${k}_{cat}$ at different temperatures, to explore the possibility of using DLTKcat to make metabolic modeling sensitive to temperature changes.

## METHODS

### Data set preparation

The data set used to construct the deep learning model was extracted from the BRENDA and SABIO-RK databases. Enzyme class (EC) number, substrate name, organism name, protein identifier (UniProt ID), enzyme type, temperature and ${k}_{cat}$ values were queried from SABIO-RK via application programming interface (API). The data in BRENDA were fetched using BRENDApyrser [23]. The canonical SMILES string [24] of the substrate, which describes the molecular structure of chemical species, was obtained by querying the PubChem compound database [25] via API. The amino acid sequence of each enzyme protein was queried from the UniProt database [26] based on the UniProt ID also via API. The sequences of wild-type (WT) enzymes were mapped directly. For mutants caused by amino acid substitutions, amino acids at mutated locations were changed based on mutation information from BRENDA and SABIO-RK. Entries with other types of mutations were removed. All API codes can be found at https://github.com/SizheQiu/DLTKcat.

After SMILE strings and amino acid sequences were obtained, the data set filtered out all redundant entries with the same SMILE string, amino acid sequence, temperature and ${k}_{cat}$ value. For entries with the same SMILE string, amino acid sequence, temperature but different ${k}_{cat}$ values, only the entry with the largest ${k}_{cat}$ value was kept, as done in Li *et al*. [16]. Finally, 4383 entries from SABIO-RK and 11 866 entries from BRENDA remained. In all, 10 556 entries’ enzymes were WTs and 5693 entries’ enzymes were mutants (Figure S1). ${k}_{cat}$ values of 87 EC (numbers) were found to have significant correlations with temperature, which covered 2430 entries (Figure S2). Considering the uneven distribution of temperature values in the data set, oversampling was performed to append two times of entries at low (*T* < 20°C) and high (*T* > 40°C) temperature ranges by randomly duplicating existing entries at those temperature ranges (Figure S3). Because previously published CPI deep learning models have shown that additional features, such as enzyme molar mass or the octanol–water partition coefficient of substrate, could not improve model performance [15, 21], the finalized data set of this study only contained SMILES strings of substrates, amino acid sequences of enzyme proteins and temperature values.

### Construction of the deep learning model

**Figure 1:**
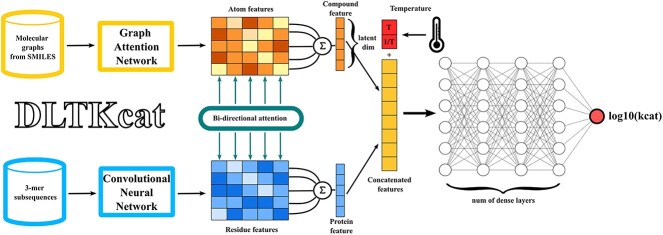
The overview of DLTKcat. With a pair of substrate and enzyme as the input, a GAT and a CNN learn the representations of the atom and residue from the compound molecular graph and protein sequence. Next, atom and residue representations are fed into the bidirectional attention neural network to integrate the representations and capture the important regions of compounds and proteins. Then, temperature ($T$) and inverse temperature ($\frac{1}{T}$) are integrated into the concatenated features. Finally, the concatenated features are used to predict the $\mathrm{lo}{\mathrm{g}}_{10}\left({k}_{cat}\right)$ value.

Similar to other CPI deep learning models, DLTKcat uses Graph Attention Network (GAT) and CNN to extract features from the substrate molecular graph and enzyme protein sequence, respectively (Figure 1). The use of bidirectional attention, adopted from BACPI by Li *et al*. [27], and integration of temperature and inverse temperature values capture the temperature-dependent interactions between atoms of the compound and residues of the protein. Finally, the concatenated features of compound, protein and temperature are fed into several dense layers (fully connected layers) to predict the $\mathrm{lo}{\mathrm{g}}_{10}\left({k}_{cat}\right)$ value.

#### Compound representation

RDKit [28] converts the SMILES string into the molecular graph of the substrate with atoms as vertices, and chemical bonds as edges. The graph, along with the initial embeddings of its vertices, is fed into the graph attentional layer of GAT. A linear learnable transformation converts the embeddings (${v}_i^{init}\in{R}^{H_c}$, ${H}_c$=80) into higher-level features of the compound (${v}_i^{`}\in{R}^{H_c^{`}}$, ${H}_c^{`}$=50). The multi-head attention mechanism in GAT concatenates output features from three independent graph attentional layers to increase the stability of the self-attention learning process. Finally, a single-layer neural network transforms concatenated features into the compound space. The final output features are atom features (${v}_i\in{R}^{H_c}$) (Figure 1). Extended Connectivity Fingerprints (ECFPs) [29] of length 1024, computed by RDKit, are also used to represent the compound. A multilayer neural network transforms ECFPs into the compound space ($f\in{R}^{H_c}$).

#### Protein representation

To capture diverse protein residue patterns, the protein sequence is split into overlapping 3-mer subsequences. 3-mer subsequences are then translated to randomly initialized embeddings (${r}_i^{init}\in{R}^{H_p}$, ${H}_p$=80). Through four convolutional layers with leaky ReLU [30] as the activation function, embeddings are transformed to higher-level features of the protein sequence that can capture the complex relationships of residues. The final output features are residue features (${r}_i\in{R}^{H_p}$) (Figure 1).

#### Bidirectional attention and integration of temperature

The bidirectional attention mechanism is used to represent the interactions between atoms of the compound and residues of the protein. Residue, atom features and fingerprints are transformed into vectors (${c}_i\in{R}^d$, ${p}_i\in{R}^d$, $hf\in{R}^d$), and a soft alignment matrix ($A\in{R}^{N_v\times{N}_r}$) indicates the interaction strengths. $d$ is the unified latent dimension ($d$ = 40, 64). The weighted information is extracted from the soft alignment matrix, and attention weights are computed in both atom-to-residue (${\alpha}_{a2r}\in{R}^{N_v}$) and residue-to-atom (${\alpha}_{r2a}\in{R}^{N_r}$) directions. The outputs are compound (${h}_c\in{R}^d$) and protein (${h}_p\in{R}^d$) features (Figure 1). To improve learning stability and representation capacity, a multi-head attention model (number of heads = 3) is used to capture diverse aspects of CPI (${h}_c^{final}\in{R}^d$, ${h}_p^{final}\in{R}^d$).

Inspired by the Arrhenius equation (${k}_{cat}=A{e}^{-\frac{E_a}{RT}}$) [20], temperature ($T$) and inverse temperature ($\frac{1}{T}$) are first normalized ($\frac{x-{x}_{min}}{x_{max}-{x}_{min}}$), and then concatenated with compound and protein features output by the bidirectional attention process. The inverse of temperature best represents the linear relationship between $\frac{1}{T}$ and $\mathrm{lo}{\mathrm{g}}_{10}\left({k}_{cat}\right)$. The concatenated features (${h}_c^{final}\left\Vert{h}_f\right\Vert{h}_p^{final}\Big\Vert \left[T,\frac{1}{T}\right] \Big\Vert$, is the concatenation operation) are then fed into several dense layers (layer number = 3–6), with leaky ReLU as the activation function, for the regression of the $\mathrm{lo}{\mathrm{g}}_{10}\left({k}_{cat}\right)$ value.

#### Model training

Because of the large size of the data set, batch training was used with a batch size of 32. Adam optimization algorithm [31] was used to update neural network weights iteratively. The loss function was mean squared error (MSE). The initial learning rate was 0.001, and the learning rate decayed by 50% for every 10 epochs to prevent overfitting. For details of software and hardware, please see Section S1.1 of the Supplementary Information.

### Interpretation of attention weights on protein residues

The bi-direction attention mechanism in section Bidirectional Attention and Integration of Temperature assigns attention weights to protein subsequences and atoms of the substrate. A higher attention weight of one residue means that residue is more important for the enzyme kinetics toward a certain substrate. The residue attention weights (${\alpha}_{r2a}$) can be computed based on the intermediate output in the deep learning model.


(1) \begin{equation*} {c}_i= LeakyReLU\left({W}_v{v}_i\right) \end{equation*}

(2) \begin{equation*} {p}_i= LeakyReLU\left({W}_r\ {r}_i\right) \end{equation*}

(3) \begin{equation*} {I}_p={A}^T\mathit{\tanh}\left(C{W}_{a2r}\right) \end{equation*}

(4) \begin{equation*} {\alpha}_{r2a}= softmax\left(\left[P{W}_p\ \Big\Vert\ {I}_p\right]{a}_{r2a}\right) \end{equation*}

${v}_i$ and ${r}_i$ are atom and residue feature vectors (sections Compound Representation and Protein Representation), ${W}_v\in{R}^{d\times{H}_c}$ and ${W}_r\in{R}^{d\times{H}_p}$ transform ${v}_i$ and ${r}_i$ to ${c}_i$ and ${p}_i$, respectively (Equations (1 and 2)). $d$ is the latent dimension in the bidirectional attention mechanism. $C=\left[{c}_1,{c}_2,\dots, {c}_{N_v}\right]$, $P=\left[{p}_1,{p}_2,\dots, {p}_{N_r}\right]$, ${W}_{a2r}\in{R}^{d\times d}$, $A=\mathit{\tanh}\left( CU{P}^T\right)\in{R}^{N_v\times{N}_r}$ is a pairwise interaction matrix for atoms and residues, and ${I}_p\in{R}^{N_v\times d}$ represents information from atoms to residues (Equation (3)). ${W}_p\in{R}^{d\times d}$, $\Vert$ is the concatenation operation, ${a}_{r2a}\in{R}^{2d}$, and the vector of residue attention weights (${\alpha}_{r2a}$) is protein attention weights normalized by the softmax function (Equation (4)).

### Proteome constrained flux balance analysis with predicted ${\boldsymbol{k}}_{\boldsymbol{cat}}$

Flux balance analysis has been used to estimate metabolic fluxes and cellular growth rates for decades [32]. The basic required inputs are the stoichiometric matrix ($S$) from the genome-scale metabolic model (GSMM) [32] and growth medium parameters that set upper bounds for nutrient uptake rates. Flux balance analysis computes metabolic fluxes (${v}_i$) by maximizing an objective function (Equation (5)), which is usually the growth function [${v}_{growth}$, biomass formation rate normalized to 1 gram dry weight (gDW) of biomass], via linear optimization in a constrained solution space of mass conservation (Equation (6)) and lower/upper bounds (${v}_{lb}$, ${v}_{ub}$) of reaction fluxes (Equation (7)). Flux balance analysis was conducted using COBRApy [33] in this study.


(5) \begin{equation*} \mathit{\operatorname{Max}}\ {v}_{growth} \end{equation*}

(6) \begin{equation*} S\ast v=0 \end{equation*}

(7) \begin{equation*} {v}_{lb}\le{v}_i\le{v}_{ub} \end{equation*}

Proteome constrained flux balance analysis tightens the solution space by integrating proteome constraints of reactions into conventional flux balance analysis [34]. The reaction flux (${v}_i$, $\frac{mmol}{hr\ast gDW}$) is constrained by the enzyme capacity (${k}_i\left[{E}_i\right]$ or ${a}_i\left(M{W}_i\ast \left[{E}_i\right]\right)$) (Equation (8)). ${k}_i$ is the ${k}_{cat}$ of reaction $i$ and $\left[{E}_i\right]$ is the enzyme molar concentration ($\frac{mmol}{gDW}$). ${a}_i$ ($\frac{\mu mol}{\mathit{\min}\ast mg\ E}$) is the enzyme-specific activity, defined as the micro moles of products formed by an enzyme in a given amount of time per milligram of the enzyme protein. $M{W}_i$ is enzyme molar mass ($\frac{g}{mol}$). Proteome was divided into sectors of inflexible housekeeping (Q), anabolism (A), transportation (T) and catabolism (C). The upper bound of all flexible sectors (i.e. C, A, T) combined was assumed to be 50% of the total proteome (Equation (9)) [35–37].


(8) \begin{equation*} {v}_i\le{k}_i\left[{E}_i\right]\ or\ {v}_i\le{a}_i\left(M{W}_i\ast \left[{E}_i\right]\right) \end{equation*}

(9) \begin{equation*} {\phi}_Q\left(50\%\right)+{\phi}_C+{\phi}_A+{\phi}_T\le 100\% \end{equation*}

(10) \begin{equation*} {\phi}_A\ast{P}_{TOT}=M{W}_{ribosome}\ast \left[{E}_{ribosome}\right]=\frac{v_{growth}}{a_{ribosome}} \end{equation*}

(11) \begin{equation*} {\phi}_C\ast{P}_{TOT}=\sum M{W}_i\ast \left[{E}_i\right]=\sum \frac{v_i\ast M{W}_i}{k_i} \end{equation*}

${\phi}_x$ is the mass fraction of sector $x$ for $x=A,C,T$. ${P}_{TOT}$ is the total mass of the proteome normalized to 1 gDW of biomass ($\frac{g}{gDW}$). The enzyme activity of the ribosome for the anabolism sector (${a}_{ribosome}$) was set as $107.4\ \frac{mmol}{hr\ast g\ E}$ (Equation (10)) [36, 38]. ${k}_{cat}$ values were predicted for the catabolic sector (sector C), by DTLKcat (Equation (11)).

In this study, proteome constrained flux balance analysis was performed for *Lactococcus lactis MG1363* (LL) and *Streptococcus thermophilus LMG18311* (ST). The GSMMs used were obtained from the work of Flahaut *et al*. [39] and Pastink *et al*. [40]. Experimental data of LL and ST’s growth rates at different temperatures were obtained from Chen *et al.* [41] and Vaningelgem *et al.* [42]. The carbon sources of LL and ST, in experiments, were glucose and lactose, respectively. Therefore, the enzyme activities (${a}_{CT}$, CT stands for carbon source transportation) of glucose transport via phosphotransferase system and lactose: galactose antiporter were set as $361.14\ \frac{mmol}{hr\ast gE}$ [43] and $540\ \frac{mmol}{hr\ast gE}$ [44] (Equation (11)). Because both lactose and glucose were sufficient in the growth medium [41, 42], no Michaelis–Menten kinetics was needed for transporter proteins. Lactic and acetic acids were two major products of the central carbon metabolism of lactic acid bacteria, and the enzyme activity of acid exportation (${a}_{AT}$) was set as $6360\ \frac{mmol}{hr\ast gE}$ [36, 38] (Equation (12)).


(12) \begin{equation*} {\phi}_T\ast{P}_{TOT}=M{W}_{AT}\ast \left[{E}_{AT}\right]+M{W}_{CT}\ast \left[{E}_{CT}\right]=\frac{v_{AT}}{a_{AT}}+\frac{v_{CT}}{a_{CT}} \end{equation*}

Temperature-dependent ${k}_{cat}$ values were predicted for enzymes in two bacteria’s central carbon metabolism (Tables S1 and S2). The SMILES strings of substrates were queried from PubChem with metabolite names in GSMMs, and protein sequences were queried from UniProt with gene locus tags in genome assemblies of LL, GCF_000009425.1 [45], and ST, GCF_000011825.1 [46]. The predicted ${k}_{cat}$ for the primary substrate of each reaction was selected as the ${k}_{cat}$ of the reaction. For isozymes that catalyze the same metabolic reaction, the largest ${k}_{cat}$ was selected. Both ST and LL are important and widely used lactic acid bacteria, but their enzyme ${k}_{cat}$ values are quite limited in databases. For example, there are only 11 entries for ST in SABIO-RK, most were contributed by Simon and Hofer [47]. Therefore, this study used DLTKcat to fill the gap and examined DLTKcat’s performance in predicting metabolic responses to temperature changes.

## RESULTS

### DLTKcat has good performance on temperature-dependent ${\mathbf{k}}_{\mathbf{cat}}$ prediction

**Figure 2:**
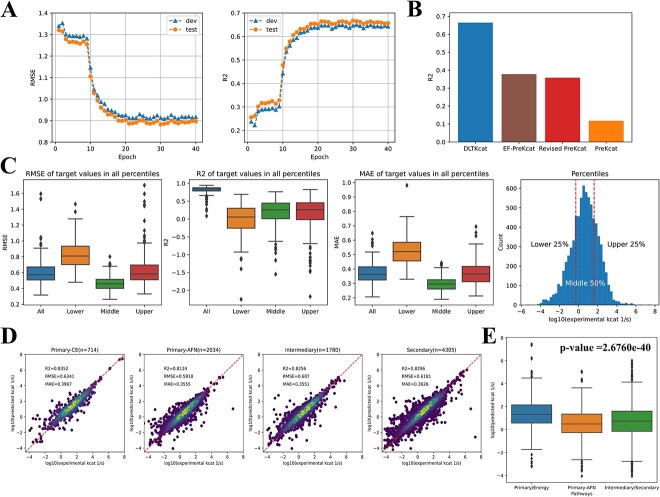
Assessment of the model performance. (**A**) The RMSE and *R*^2^ scores of $\mathrm{lo}{\mathrm{g}}_{10}\left({k}_{cat}\right)$ prediction during the training process. Test: the test set; dev: the validation set. The RMSE and *R*^2^ of the test set at the end of training are 0.88 and 0.66. (**B**) Comparison of reported *R*^2^ scores of DLTKcat, EF-UniKP, Revised UniKP and UniKP on $\mathrm{lo}{\mathrm{g}}_{10}\left({k}_{cat}\right)$ prediction with temperature values. (**C**) The distributions of RMSE, *R*^2^ and MAE scores of $\mathrm{lo}{\mathrm{g}}_{10}\left({k}_{cat}\right)$ prediction for target values at lower 25%, middle 50% and upper 25% percentiles. (**D**) *R*^2^, RMSE and MAE scores of $\mathrm{lo}{\mathrm{g}}_{10}\left({k}_{cat}\right)$ prediction for enzymes in primary-CE, primary-AFN, intermediary and secondary metabolism. (**E**) The comparison of distributions of predicted $\mathrm{lo}{\mathrm{g}}_{10}\left({k}_{cat}\right)$ values in primary-CE and other metabolic pathways (*P*-value < 0.001). Primary-AFN, primary metabolism—amino acid/fatty acid/nucleotide.

With optimal hyperparameters (Section S1.3 and Figure S4), the model training process reduced RMSE (Equation (S2)) scores of predicted $\mathrm{lo}{\mathrm{g}}_{10}\left({k}_{cat}\right)$ of the test data set from 1.33 to 0.88, and enhanced *R*^2^ (Equation (S1)) scores from 0.25 to 0.66 after 20 epochs (Figure 2A). The *R*^2^ scores of previously published deep learning models on temperature-dependent ${k}_{cat}$ were all reported to be below 0.5 [22], and DLTKcat has outperformed them by reaching a *R*^2^ score of 0.66 on the randomly selected test data set (Figure 2B). In addition, DLTKcat showed good prediction accuracy with low RMSE and mean absolute error (MAE; Equation (S3)) scores for sub-data sets with experimental $\mathrm{lo}{\mathrm{g}}_{10}\left({k}_{cat}\right)$ values at the lower 25%, middle 50% and upper 25% ranges (Figure 2C). In a nutshell, DLTKcat demonstrated superior performance in comparison to previously published deep learning models for temperature-dependent ${k}_{cat}$, and a robust accuracy for target values [experimental $\mathrm{lo}{\mathrm{g}}_{10}\left({k}_{cat}\right)$ values] at different ranges.

To explore the predictive power of DLTKcat across different metabolic contexts, the prediction accuracy of $\mathrm{lo}{\mathrm{g}}_{10}\left({k}_{cat}\right)$ values for enzymes in four different pathways, categorized based on enzyme modules in KEGG database [48], were assessed, and *R*^2^, RMSE and MAE scores were all around 0.8, 0.6 and 0.35 (Figure 2D). After the validation of DLTKcat’s good accuracy across different metabolic contexts, the model showed its ability to discriminate enzymes in primary metabolism—catabolism/energy (primary-CE) and other pathways, with higher predicted $\mathrm{lo}{\mathrm{g}}_{10}\left({k}_{cat}\right)$ values in primary-CE (*P*-value < 0.001) (Figure 2E). In short, DLTKcat could well characterize enzymes from different metabolic contexts.

### Interpretation of ${\boldsymbol{k}}_{\boldsymbol{cat}}$ prediction of mutated enzymes

**Figure 3:**
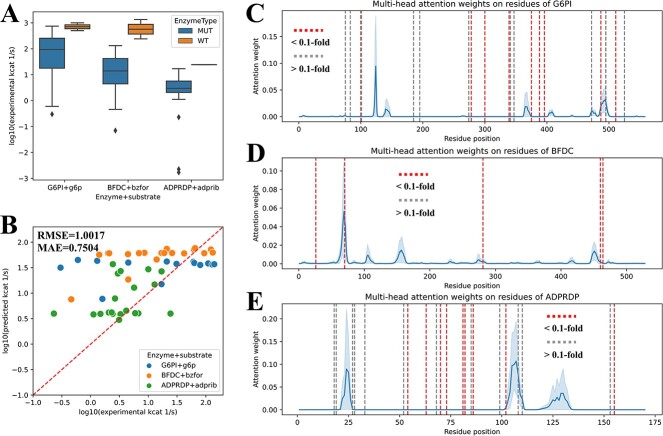
DLTKcat for the prediction and interpretation of ${k}_{cat}$ of mutated enzymes. (**A**) The comparison between experimental $\mathrm{lo}{\mathrm{g}}_{10}\left({k}_{cat}\right)$ of WT and mutated enzymes for G6PI + g6p, BFDC + bzfor and ADPRDP + adprib. MUT, mutant. (**B**) RMSE and MAE scores of predicted $\mathrm{lo}{\mathrm{g}}_{10}\left({k}_{cat}\right)$ values for G6PI + g6p, BFDC + bzfor and ADPRDP + adprib. RMSE = 1.0017, MAE = 0.7504. (**C**) Multi-head attention weights on residues of the WT G6PI and mutation sites. (**D**) Multi-head attention weights on residues of the WT BFDC and mutation sites. (**E**) Multi-head attention weights on residues of the WT ADPRDP and mutation sites. Dark dash line: mutation site (<0.1-fold WT ${k}_{cat}$); pale dash line: mutation site (>0.1-fold WT ${k}_{cat}$); solid curve: attention weight.

First, the accuracy of DLTKcat for both WT and mutated enzymes was examined, and *R*^2^, RMSE and MAE scores were around 0.8, 0.6 and 0.4, respectively (Figure S5). After the prediction accuracy was ensured, this study selected three enzyme–substrate pairs with more than 20 mutations in the data set to investigate how DLTKcat captures amino acid substitutions. The three enzyme–substrate pairs were glucose-6-phosphate isomerase and D-glucose 6-phosphate (G6PI + g6p), benzoylformate decarboxylase and benzoylformate (BFDC + bzfor) and ADP-ribose diphosphatase and ADP-ribose (ADPRDP + adprib). The uniprot IDs of three enzyme proteins wereP06744, P20906 and Q5SKW5. Amino acid substitutions on protein sequences of three enzymes all resulted in the decrease of ${k}_{cat}$ (Figure 3A). The prediction accuracy of the selected three enzyme–substrate pairs was slightly lower than that of all mutated enzymes, but the prediction error was still around one order of magnitude (Figures 3B and S5). Next, the mapping of mutation sites to residue attention weights (section Interpretation of Attention Weights on Protein Residues) shows that most mutation sites (<0.1-fold WT ${k}_{cat}$) distribute closely to peaks of attention weights (Figure 3C–E). The overlapping between mutation sites (<0.1-fold WT ${k}_{cat}$) and residues with high attention weights was most noticeable for residue 70, 460 and 464 on BFDC (Figure 3D). Generally speaking, DLTKcat is a good predictor for mutated enzymes, and residue attention weights can reflect the impact of amino acid substitutions on enzyme kinetics.

### The contribution of temperature-related features to ${k}_{cat}$ prediction

**Figure 4:**
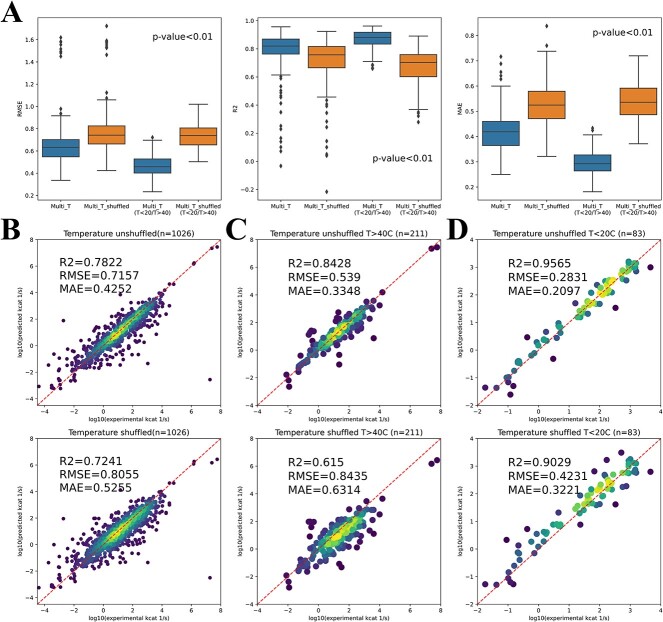
The importance of temperature-related features in DLTKcat. (**A**) The distributions of RMSE, *R*^2^, MAE scores of predicted $\mathrm{lo}{\mathrm{g}}_{10}\left({k}_{cat}\right)$ values with unshuffled and shuffled temperature-related features for the selected data set with 1026 entries (Multi_T) and for entries of low (*T* < 20°C) and high (*T* > 40°C) temperature. (**B**) *R*^2^, RMSE and MAE scores of predicted $\mathrm{lo}{\mathrm{g}}_{10}\left({k}_{cat}\right)$ values with unshuffled and shuffled temperature-related features for the selected data set with 1026 entries. (**C**) *R*^2^, RMSE and MAE scores of predicted $\mathrm{lo}{\mathrm{g}}_{10}\left({k}_{cat}\right)$ values with unshuffled and shuffled temperature-related features for entries of high temperature. (**D**) *R*^2^, RMSE and MAE scores of predicted $\mathrm{lo}{\mathrm{g}}_{10}\left({k}_{cat}\right)$ values with unshuffled and shuffled temperature-related features for entries of low temperature.

Before feature importance analysis, the prediction accuracy was examined for different temperature ranges (below 20°C, above 40°C and between 20 and 40°C). High *R*^2^ and low RMSE scores reflected that DLTKcat could accurately predict ${k}_{cat}$ for low, middle and high temperatures, with an error far below one order of magnitude (Figure S6). Then, feature shuffling, also known as feature permutation, was performed to show the importance of temperature and inverse temperature values (Section S1.4). The shuffling of temperature features resulted in significantly higher distributions of the prediction error (RMSE and MAE), and lower distributions of *R*^2^ than those of predictions with unshuffled temperature features (Figure 4A). The comparison between predicted and experimental values showed that the RMSE and MAE scores increased by around 0.1 and *R*^2^ decreased by around 0.1 when temperature-related features were shuffled (Figure 4B). For high (*T* > 40°C) and low (*T* < 20°C) temperature ranges, the increase of RMSE and MAE and decrease of *R*^2^, caused by feature shuffling became larger (Figure 4C and D). In short, the decrease in prediction accuracy with shuffled temperature-related features demonstrated the importance of temperature-related features in DLTKcat.

### Use DLTKcat to predict ${\boldsymbol{k}}_{\boldsymbol{cat}}$ of WT and mutated *Pyrococcus furiosus* Ornithine Carbamoyltransferases

**Figure 5:**
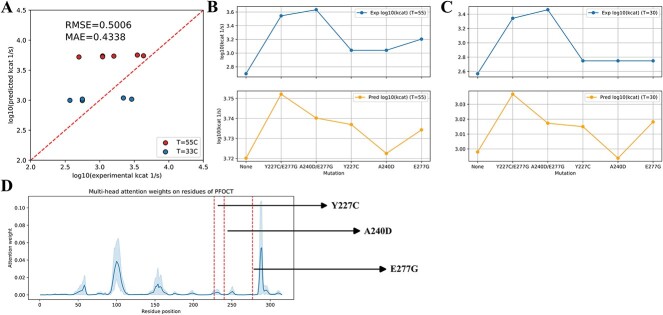
Prediction of the effect of amino acid substitutions on ${k}_{cat}$ values. (**A**) Comparison between experimental and predicted $\mathrm{lo}{\mathrm{g}}_{10}\left({k}_{cat}\right)$ of *P. furiosus* ornithine carbamoyltransferase, RMSE = 0.5006, MAE = 0.4338. (**B**) Experimental (Exp) and predicted (Pred) $\mathrm{lo}{\mathrm{g}}_{10}\left({k}_{cat}\right)$ values of WT and mutants at 55°C. (**C**) Experimental (Exp) and predicted (Pred) $\mathrm{lo}{\mathrm{g}}_{10}\left({k}_{cat}\right)$ values of WT and mutants at 30°C. Exp, experimental value; Pred, predicted value. (**D**) Multi-head attention weights on residues of the WT *P. furiosus* ornithine carbamoyltransferase protein sequence. Dash-line: mutation site.

The ${k}_{cat}$ values of WT and mutated *Pyrococcus furiosus* ornithine carbamoyltransferases at 30 and 55°C were obtained from Roovers *et al*. [49]. The protein sequence of *P. furiosus* ornithine carbamoyltransferase was obtained from Uniprot with the Uniprot ID of Q51742. The prediction achieved high accuracy (RMSE = 0.5, MAE = 0.4338) (Figure 5A). Predicted ${k}_{cat}$ values at 55°C were higher than those at 30 C (Figure 5A–C), which was both consistent with the experimental data and the nature of *P. furiosus* being a hyperthermophile favoring high temperature [50].

With respect to the effect of mutations, DLTKcat suggested that amino acid substitutions at 227th, 240th and 277th amino acids could increase the ${k}_{cat}$ value, consistent with the experimental data, despite that the numerical difference between predicted ${k}_{cat}$ values of mutants and WT was small (Figure 5B and C; note the difference in scale between upper and lower *y*-axes). Furthermore, DLTKcat also captured that the combination of two amino acid substitutions, Y227C/E277G and A240D/E277G, could result in greater improvement on the ${k}_{cat}$ value than the substitution at each single site, though it failed to predict that the ${k}_{cat}$ of A240D/E277G was higher than that of Y227C/E277G (Figure 5B and C). The mapping of mutation sites to residue attention weights showed that E277G, as the mutation with a higher enhancement of ${k}_{cat}$ than other two mutations, was also closer to the high peak of attention weights (Figure 5D). In addition, residue attention weights indicated other potential mutation sites on *P. furiosus* ornithine carbamoyltransferase that might have substantial effects on ${k}_{cat}$ (Figure 5D).

### Temperature sensitive metabolic modeling with predicted ${\boldsymbol{k}}_{\boldsymbol{cat}}$

**Figure 6:**
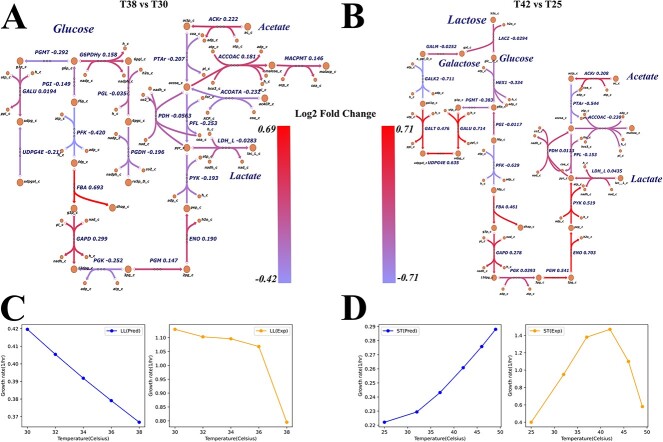
Prediction of bacteria metabolism at different temperatures. (**A**) Log2-fold change of predicted ${k}_{cat}$ values for LL at 38 and 30°C (38 versus 30°C). (**B**) Log2-fold change of predicted ${k}_{cat}$ values for ST at 42 and 25°C (42 versus 25°C). (**C**) Comparison of predicted (Pred) and experimental (Exp) growth rates of LL at 30, 32, 34, 36 and 38 °C. (**D**) Comparison of predicted (Pred) and experimental (Exp) growth rates of ST at 25, 32, 37, 42, 46 and 49°C. Exp, experimental value; Pred, predicted value. Reaction information can be found in Tables S1 and S2.

DLTKcat predicted ${k}_{cat}$ values for enzymes of LL at 30, 32, 34, 36 and 38°C, and of ST at 25, 32, 37, 42, 46 and 49°C, which were temperatures where LL and ST’s growth rates were measured in experimental data [41, 42]. DLTKcat predicted that ${k}_{cat}$ of most catabolic enzymes in LL would decrease when temperature increased from 30 to 38°C, especially for G6PI (PGI), phosphofructokinase (PFK), phosphoglycerate kinase (PGK), pyruvate kinase (PYK), pyruvate formate lyase (PFL) and phosphotransacetylase (PTAr) (Figure 6A). The predicted decrease of the activity of catabolism in LL in response to temperature increase is consistent with the experimental observation that LL stopped growing after temperature became larger than 38°C [41]. For catabolic enzymes in ST, DLTKcat predicted that most enzymes’ ${k}_{cat}$ would increase when temperature increased from 25 to 42°C, especially for fructose-bisphosphate aldolase (FBA, not the abbreviation of flux balance analysis), Glyceraldehyde-3-phosphate dehydrogenase (GAPD), phosphoglycerate mutase (PGM), enolase (ENO) and pyruvate kinase (PYK) (Figure 6B). The predicted increase of catabolic activity in ST when temperature increases to 42°C is consistent with both the experimental data [42] and the nature of ST being a thermophile [51]. These results showed that, in general, DLTKcat could qualitatively predict metabolic responses of bacteria to certain temperature changes.

However, the quantitative accuracy of growth rates computed by proteome constrained flux balance analysis was low. In proteome constrained flux balance analysis for LL, the ${k}_{cat}$ of fructose-bisphosphate aldolase (FBA) in LL was fixed at $13.9\ \frac{1}{s}$ [52] in sacrifice of temperature sensitivity, because predicted ${k}_{cat}$ values at different temperatures were unrealistically low ($0.04\sim 0.065\ \frac{1}{s}$), compared with experiment ${k}_{cat}$ values in other bacteria [52, 53]. The predicted growth rates of LL by proteome constrained flux balance analysis captured the decreasing trend in response to the increase of temperature, but the predicted values were deviant from experimental values (Figure 6C). The proteome constrained flux balance analysis predicted the increase of ST’s growth rate from 25 to 42°C, but it failed to predict the drop of growth rate from 42 to 49°C (Figure 6D). Also, the predicted increase of ${k}_{cat}$ values from 42 to 49°C by DLTKcat (Figure S7) contradicted the experimental finding that 49°C is close to the theoretical maximum temperature for ST to survive, 47–50°C [51]. To conclude, the log10-scale RMSE score within 1 of DLTKcat is not low enough to enable temperature sensitive proteome constrained flux balance analysis to predict bacterial growth and metabolism with good quantitative accuracy.

## DISCUSSION

The expensive cost of obtaining enzyme ${k}_{cat}$ values in wet lab stimulates the need of developing computational models to predict ${k}_{cat}$. Nevertheless, predicting temperature-dependent ${k}_{cat}$ is a challenging task, as temperature is not only a variable in the exponential factor of the Arrhenius equation, it also affects the activation energy of the enzyme catalyzed reaction, which is governed by the CPI [20]. To tackle the challenging task, this study constructed a CPI deep learning model called DLTKcat. DLTKcat used the bidirectional attention mechanism [27] to represent the interactions between compounds and proteins, and attention weights could capture important regions on protein sequences (section Interpretation of ${k}_{cat}$ Prediction of Mutated Enzymes). The use of both temperature and inverse temperature values facilitated the learning process of the neural network by representing features in the most biophysical relevant form to ${k}_{cat}$ [20]. Also, oversampling on entries at low and high temperature ranges compensated for the imbalanced distribution of temperature values in the data set (Figure S3). As a result, DLTKcat showed superior performance (log10-scale RMSE = 0.88, *R*^2^ = 0.66) than previously published models (e.g. EF-UniKP) and robust accuracy for ${k}_{cat}$ predictions for different conditions (e.g. metabolic contexts). In addition, feature shuffling demonstrated the contribution of temperature-related features to this deep learning model.

By accurately predicting the effect of protein sequence mutations on the ${k}_{cat}$ value of *P. furiosus* ornithine carbamoyltransferase at different temperatures (section Use DLTKcat to Predict ${k}_{cat}$ of WT and Mutated *P. furiosus* Ornithine Carbamoyltransferases), DLTKcat exhibited its function in scoring the efficiency of in silico designed enzyme protein sequences. Imaginably, the combination of DLTKcat and optimization algorithms (e.g. genetic programming) can become a computational tool to design site-specific mutagenesis to optimize enzyme catalysis, which will be more efficient than directed evolution that relies on random mutagenesis.

Nonetheless, the second case study (section Temperature Sensitive Metabolic Modeling with Predicted ${k}_{cat}$) of generating temperature-dependent proteome constraints for metabolic modeling revealed the limitation of DLTKcat that its prediction error was not low enough to accurately model the response of cellular metabolism to temperature changes. Because all ${k}_{cat}$ values of catabolic enzymes in ST and LL were predicted by DLTKcat, the propagation of error led to the inaccuracy of proteome constrained flux balance analysis. In short, deep learning can gap fill a few missing ${k}_{cat}$ values in the metabolic network, as done in Li *et al*. [16], but the accuracy of proteome constrained flux balance analysis will not be high if most proteome constraints are based on predicted ${k}_{cat}$ values.

To further improve the performance and utility of DLTKcat, including additional experimental conditions like pH, metal ion concentrations might be an approach, but the lack of data restricted existing models from accounting for those factors [22]. Including the optimal enzyme temperature either from databases or predictions [54] might be able to enhance the temperature sensitivity of DLTKcat. The difference between the experimental temperature and optimal temperature could inform the model whether the temperature feature has a negative or positive effect on the ${k}_{cat}$ value. However, the success of this approach depends on the accuracy of enzyme optimal temperature prediction, which was reported to have a RMSE around 2 [54, 55].

Overall, DLTKcat can provide accurate predictions of ${k}_{cat}$ and account for the effect of temperature changes. Two case studies (3.4 Use DLTKcat to predict k_cat_ of wild-type and mutated Pyrococcus furiosus Ornithine Carbamoyltransferases and 3.5 Temperature sensitive metabolic modeling with predicted k_cat_) have revealed potential applications of DLTKcat on protein engineering, bacterial phenotype prediction, etc. Additionally, DLTKcat can be easily modified to predict other temperature-dependent CPIs, such as ${K}_m$ [56, 57]. With future improvements of the model framework, DLTKcat, as we envisage, will become a computational tool to quantitatively model the temperature dependence of biological systems, and contribute to the development of bioprocess digital twins.

**Key Points**

- This study constructed a deep learning model, DLTKcat, to predict temperature-dependent enzyme ${k}_{cat}$ with superior accuracy.
- The feature importance of temperature in predicting enzyme ${k}_{cat}$ was validated.
- DLTKcat can predict enzyme ${k}_{cat}$ for WT and mutated enzymes under different temperatures with good accuracy.
- With predicted enzyme ${k}_{cat}$ under different temperatures, proteome constrained flux balance analysis has the potential to model temperature-dependent cellular metabolism.

## Supplementary Material

SI_DL_rev_bbad506

## Data Availability

The code and data are openly available at https://github.com/SizheQiu/DLTKcat.

## ABBREVIATION

ADPRDP: ADP-ribose diphosphatase

API: application programming interface.

BFDC: benzoylformate decarboxylase.

CNN: convolutional neural network.

CPI: compound–protein interaction.

ECFP: Extended Connectivity Fingerprint.

GAT: graph attention network.

G6PI: glucose-6-phosphate isomerase.

gDW: gram dry weight.

GNN: graph neural network.

LL: *Lactococcus lactis MG1363.*

Leaky ReLU: leaky Rectified Linear Unit.

MAE: mean absolute error.

MSE: mean squared error.

MUT: mutant.

*R* ^2^: *R*-squared, the coefficient of determination.

RMSE: root mean squared error.

RNN: recurrent neural network.

SMILES: simplified molecular-input line-entry system.

ST: *Streptococcus thermophilus LMG18311.*

WT: wild type.

## Author Biographies

**Sizhe Qiu** is a PhD student at the Department of Engineering Science, University of Oxford. His research interests are in bacterial metabolism, bioinformatics and deep learning.

**Simiao Zhao** is a PhD student of Radcliffe department of Medicine, University of Oxford. His research interests include applying and developing machine learning tools to analyze biological data.

**Aidong Yang** is a Professor of Engineering Science at the Department of Engineering Science, University of Oxford. His research interests include mathematical modelling of biological systems such as metabolic networks and microbial communities.
