# Supplementary material for: DLTKcat: deep learning-based prediction of temperature-dependent enzyme turnover rates
Source: Brief Bioinform. 2024 Jan 6;25(1):bbad506. doi: 10.1093/bib/bbad506 (PMC10772988; doi:10.1093/bib/bbad506)
Supplement: SI_DL_rev_bbad506 [file si_dl_rev_bbad506.docx]

**DLTKcat**: deep learning based prediction of temperature dependent enzyme turnover rates

## Authorship

Sizhe Qiu^1^, Simiao Zhao^2^, Aidong Yang^1^*

^1^Department of Engineering Science, University of Oxford, OX1 3PJ, United Kingdom

^2^Radcliffe Department of Medicine, University of Oxford, OX3 9DU, United Kingdom

*Corresponding author: [aidong.yang@eng.ox.ac.uk](mailto:aidong.yang@eng.ox.ac.uk) (A. Yang)

## 1. Supplementary methods

### 1.1 Software and code availability

All scripts were written in python. The deep learning model was implemented using PyTorch v1.7.1. The computer used in this work was a Dell Latitude Laptop with intel core i7 CPU. The model was trained with GPU RTX8000 provided by Advanced Research Computing (ARC) service in the University of Oxford [[1]](https://paperpile.com/c/0lMnu7/Vz97). Figures were edited using InkScape (<https://inkscape.org/>). The code and data used to generate results of this paper are available at <https://github.com/SizheQiu/DLTKcat>.

### 1.2 Deep learning model evaluation

The original dataset was randomly split into the test and the train dataset with a ratio of 1:9. The test dataset was held out to examine the accuracy of the model. Before the training, 10% of the train dataset was randomly split as the validation dataset (also called dev set). During the training process, R2 (Eq. S1) and RMSE (Eq. S2) scores of $k_{cat}$ predictions were computed at each epoch for the test and validation datasets.

$R^{2}=\frac{\sum_{i=1}^{n} (y_{ie}-y_{ip})^{2}}{\sum_{i=1}^{n} (y_{ie}-\underline{y})^{2}} (Eq. S1)$

$RMSE=\sqrt{\frac{1}{n}\sum_{i=1}^{n} (y_{ie}-y_{ip})^{2}} (Eq. S2)$

For distributions of prediction errors for a certain sub-dataset, this study randomly selected 50 data entries for 200 times to obtain the distribution of RMSE (Eq. S2), R2 (Eq. S1) and mean absolute error (MAE, Eq. S3).

$MAE = \frac{1}{n}\sum_{i=1}^{n} \left| y_{ie}-y_{ip} \right| (Eq. S3)$

### 1.3 Hyperparameter optimization for latent dimension and number of dense layers

To find the optimal combinations of the latent dimension, the dimension of protein and compound features in bi-directional attention process, and the number of dense layers after feature concatenation (**see main text, Figure 1**), training processes were performed for latent dimension = 40, 64 and number of dense layers = 3, 4, 5, 6. The latent dimension and number of dense layers that achieved the lowest RMSE score on the dev set were used to generate the trained deep learning model (**see SI, Figure S4**). The other hyperparameters were reported in section 2.2 in the main text.

### 1.4 Assessment of the feature importance of temperature by random shuffling

1026 entries in the curated dataset had the same protein sequence and substrate as other entries but different temperatures. To assess the feature importance of temperature, they were extracted to obtain R2 (Eq. S1), RMSE (Eq. S2) and MAE (Eq. S3) scores of $k_{cat}$ predictions with shuffled and unshuffled temperature related features. The feature shuffling was performed by randomly sampling from the original list of feature values, so that the shuffled and unshuffled features still had the same distribution.

## 2. Tables

| Table S1. Metabolic reaction information of *Lactococcus lactis MG1363* | | |
| --- | --- | --- |
| ID | name | EC number |
| GLCpts | D-glucose transport via PEP:Pyr phosphotransferase system | _ |
| G6PDH | Glucose-6-phosphate dehydrogenase | 1.1.1.49 |
| PGL | 6-phosphogluconolactonase | 3.1.1.31 |
| PGDH | 6-phosphogluconate dehydrogenase | 1.1.1.351, 1.1.1.44 |
| GALU | UTP-glucose-1-phosphate uridylyltransferase | 2.7.7.9 |
| PGMT | Phosphoglucomutase | 5.4.2.2, 5.4.2.5 |
| UDPG4E | UDPglucose 4-epimerase | 5.1.3.2 |
| PGI | Glucose-6-phosphate isomerase | 5.3.1.9 |
| PFK | Phosphofructokinase | 2.7.1.11 |
| FBA | Fructose-bisphosphate aldolase | 4.1.2.13 |
| TPI | Triose-phosphate isomerase | 5.3.1.1 |
| GAPD | Glyceraldehyde-3-phosphate dehydrogenase | 1.2.1.12 |
| PGK | Phosphoglycerate kinase | 2.7.2.3 |
| PGM | Phosphoglycerate mutase | 5.4.2.11 |
| ENO | Enolase | 4.2.1.11 |
| PYK | Pyruvate kinase | 2.7.1.40 |
| LDH | Lactate dehydrogenase | 1.1.1.27 |
| PFL | Pyruvate formate lyase | 2.3.1.54 |
| PDH | Pyruvate dehydrogenase | 1.2.7.1 |
| PTAr | Phosphotransacetylase | 2.3.1.8 |
| ACKr | Acetate kinase | 2.7.2.1 |
| ACCOAC | Acetyl-CoA carboxylase | 6.4.1.2 |
| MACPMT | Malonyl CoAacyl carrier protein S malonyltransferase | 2.3.1.39 |

| Table S2. Metabolic reaction information of *Streptococcus thermophilus LMG18311* | | |
| --- | --- | --- |
| ID | name | EC number |
| LCTSGALex | Lactose galactose exchange via antiporter | _ |
| LACZ | Beta-galactosidase | 3.2.1.23 |
| GALK | Galactokinase | 2.7.1.6 |
| HEX | Hexokinase (D-glucose:ATP) | 2.7.1.2 |
| GALK | Galactokinase | 2.7.1.6 |
| GALM | Aldose 1-epimerase | 5.1.3.3 |
| GALT | Galactose 1 phosphate uridylyltransferase | 2.7.7.10 |
| GALU | UTP-glucose-1-phosphate uridylyltransferase | 2.7.7.9 |
| PGMT | Phosphoglucomutase | 5.4.2.2, 5.4.2.5 |
| UDPG4E | UDPglucose 4-epimerase | 5.1.3.2 |
| PGI | Glucose-6-phosphate isomerase | 5.3.1.9 |
| PFK | Phosphofructokinase | 2.7.1.11 |
| FBA | Fructose-bisphosphate aldolase | 4.1.2.13 |
| TPI | Triose-phosphate isomerase | 5.3.1.1 |
| GAPD | Glyceraldehyde-3-phosphate dehydrogenase | 1.2.1.12 |
| PGK | Phosphoglycerate kinase | 2.7.2.3 |
| PGM | Phosphoglycerate mutase | 5.4.2.11 |
| ENO | Enolase | 4.2.1.11 |
| PYK | Pyruvate kinase | 2.7.1.40 |
| LDH | Lactate dehydrogenase | 1.1.1.27 |
| PFL | Pyruvate formate lyase | 2.3.1.54 |
| PDH | Pyruvate dehydrogenase | 1.2.7.1 |
| PTAr | Phosphotransacetylase | 2.3.1.8 |
| ACKr | Acetate kinase | 2.7.2.1 |
| ACCOAC | Acetyl-CoA carboxylase | 6.4.1.2 |

Reaction IDs, names, and enzyme EC numbers are all obtained from the BIGG database (<http://bigg.ucsd.edu/>) [[2]](https://paperpile.com/c/0lMnu7/4ZNj).

## 3. Figures


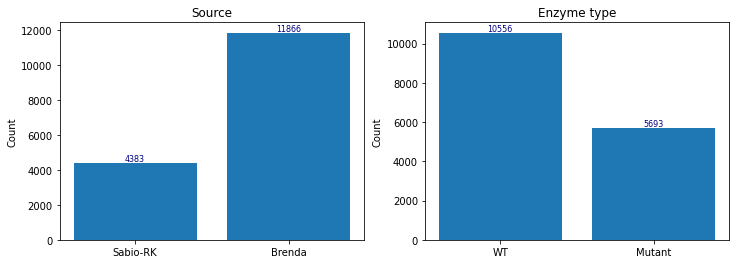


Figure S1. statistics of collected data from enzyme databases. Left: 4383 entries are from Sabio-RK, 11866 entries are from Brenda. Right: enzymes in 10556 entries are wild types, in 5693 entries are mutants.


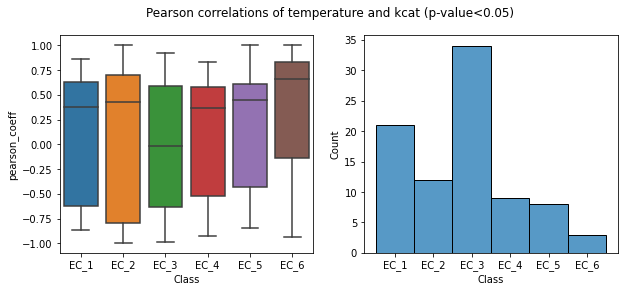


Figure S2. Significant Pearson correlations of $k_{cat}$ of 87 enzyme classes (EC numbers) covering 2430 entries and temperature.


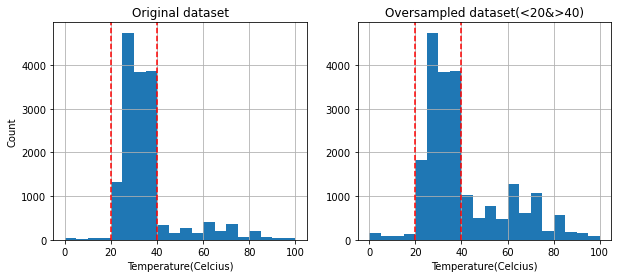


Figure S3. Training datasets before and after oversampling of entries at low and high temperature ranges.


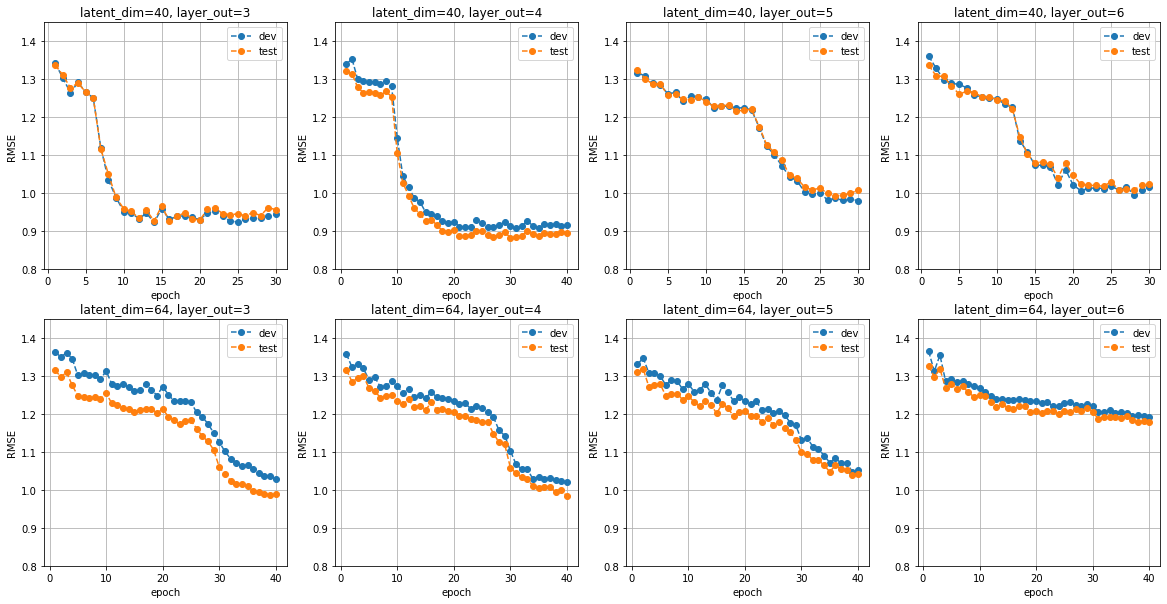


Figure S4. Results of hyperparameter optimization for latent dimension (latent_dim) and number of dense layers (layer_out). The trial with latent_dim=40 and layer_out=4 achieved the lowest RMSE. test: the test set; dev: the validation set (the development set).


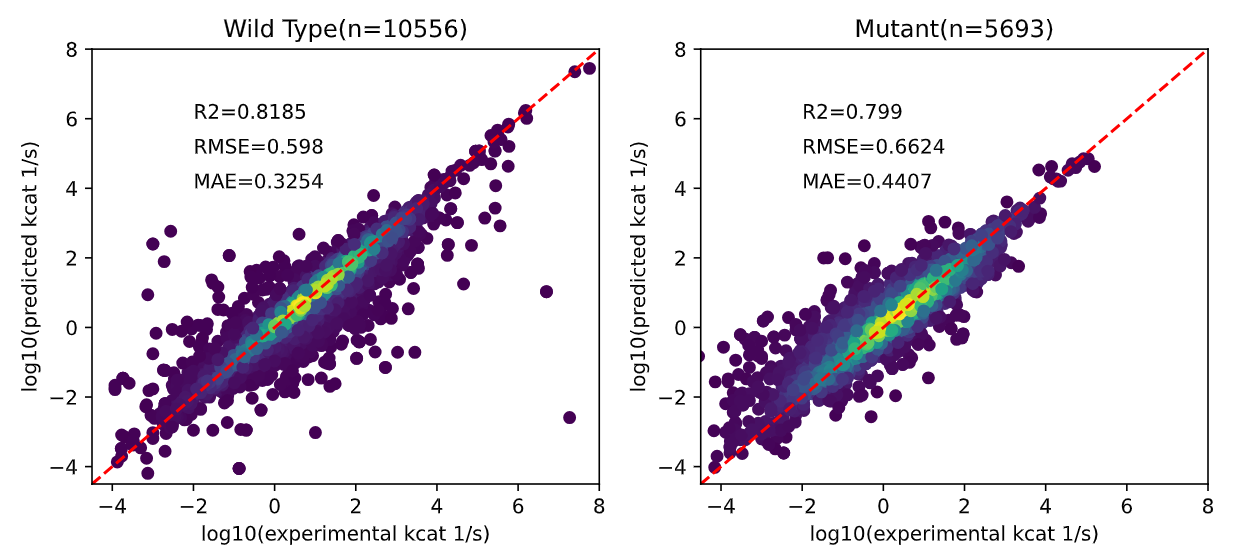


Figure S5. R2, RMSE and MAE scores of $log_{10}(k_{cat})$ prediction for WT and mutated enzymes.


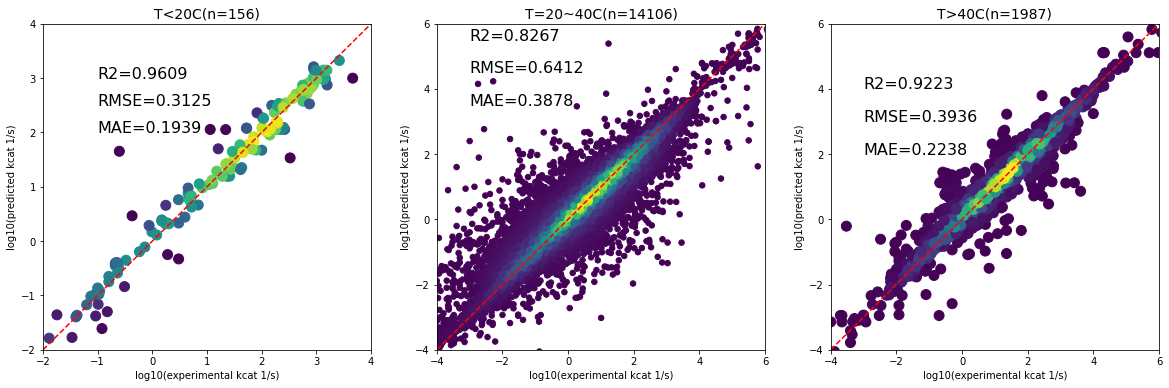


Figure S6. RMSE, R2, MAE scores of predicted $log_{10}(k_{cat})$ values at low (<20℃), middle (20~40℃) and high (>40℃) temperature ranges.


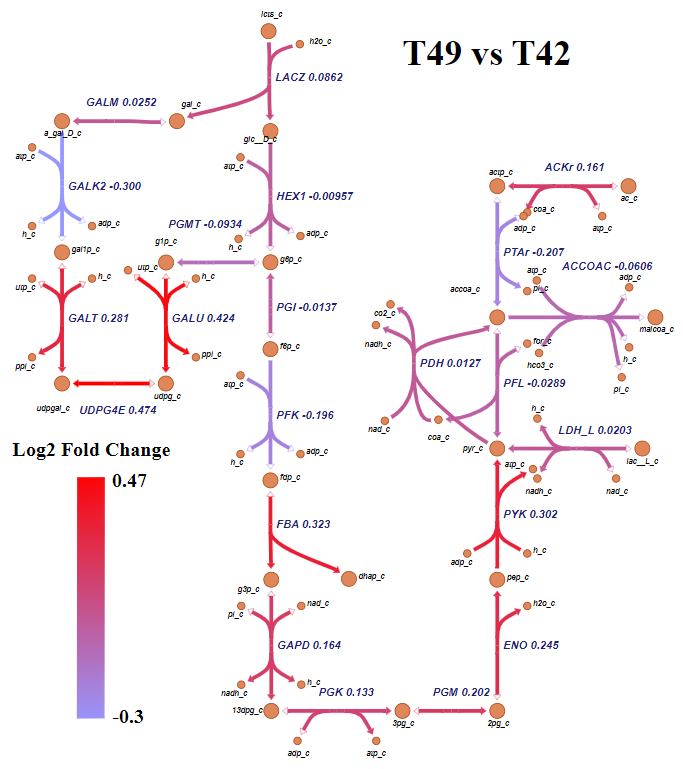


Figure S7. Log 2 fold change of predicted $k_{cat}$ values for ST at 49 ℃ and 42 ℃ (49 ℃ vs 42 ℃).

## References

[1. Richards A. University of Oxford Advanced Research Computing. 2015;](http://paperpile.com/b/0lMnu7/Vz97)

[2. King ZA, Lu J, Dräger A, et al. BiGG Models: A platform for integrating, standardizing and sharing genome-scale models. Nucleic Acids Res. 2016; 44:D515–22](http://paperpile.com/b/0lMnu7/4ZNj)
